# Acute Effects of Cheddar Cheese Consumption on Circulating Amino Acids and Human Skeletal Muscle

**DOI:** 10.3390/nu13020614

**Published:** 2021-02-13

**Authors:** Naomi M.M.P. de Hart, Ziad S. Mahmassani, Paul T. Reidy, Joshua J. Kelley, Alec I. McKenzie, Jonathan J. Petrocelli, Michael J. Bridge, Lisa M. Baird, Eric D. Bastian, Loren S. Ward, Michael T. Howard, Micah J. Drummond

**Affiliations:** 1 Department of Nutrition and Integrative Physiology, University of Utah, 250 S 1850 E, Salt Lake City, UT 84112, USA; Naomi.DeHart@utah.edu; 2 Department of Physical Therapy and Athletic Training, University of Utah, 520 Wakara Way, Salt Lake City, UT 84108, USA; Ziad.Mahmassani@health.utah.edu (Z.S.M.); joshua.kelley@utah.edu (J.J.K.); jonathan.petrocelli@utah.edu (J.J.P.); 3 Department of Kinesiology, Nutrition and Health, Miami University, 420 S Oak St., Oxford, OH 45056, USA; reidypt@miamioh.edu; 4 Geoge E. Wahlen Department of Veterans Affairs Medical Center, Geriatric Research, Education, and Clinical Center, 500 Foothill Dr., Salt Lake City, UT 84148, USA; alec.mckenzie@utah.edu; 5 Cell Imaging Facility, University of Utah, 30 N 2030 E, Salt Lake City, UT 84112, USA; Mike.Bridge@m.cc.utah.edu; 6 Department of Human Genetics, 15 N 2030 E, Salt Lake City, UT 84112, USA; lbaird@genetics.utah.edu (L.M.B.); mhoward@genetics.utah.edu (M.T.H.); 7 Dairy West Innovation Partnerships, 195 River Vista Place #306, Twin Falls, ID 83301, USA; ebastian@dairywest.com; 8 Glanbia Nutritionals Research, 450 Falls Avenue #255, Twin Falls, ID 83301, USA; LWARD@glanbia.com

**Keywords:** dairy, ribo-seq, muscle protein synthesis, anabolism, insulin

## Abstract

Cheddar cheese is a protein-dense whole food and high in leucine content. However, no information is known about the acute blood amino acid kinetics and protein anabolic effects in skeletal muscle in healthy adults. Therefore, we conducted a crossover study in which men and women (*n* = 24; ~27 years, ~23 kg/m^2^) consumed cheese (20 g protein) or an isonitrogenous amount of milk. Blood and skeletal muscle biopsies were taken before and during the post absorptive period following ingestion. We evaluated circulating essential and non-essential amino acids, insulin, and free fatty acids and examined skeletal muscle anabolism by mTORC1 cellular localization, intracellular signaling, and ribosomal profiling. We found that cheese ingestion had a slower yet more sustained branched-chain amino acid circulation appearance over the postprandial period peaking at ~120 min. Cheese also modestly stimulated mTORC1 signaling and increased membrane localization. Using ribosomal profiling we found that, though both milk and cheese stimulated a muscle anabolic program associated with mTORC1 signaling that was more evident with milk, mTORC1 signaling persisted with cheese while also inducing a lower insulinogenic response. We conclude that Cheddar cheese induced a sustained blood amino acid and moderate muscle mTORC1 response yet had a lower glycemic profile compared to milk.

## 1. Introduction

Aminoacidemia from the digestion of protein sources is a major stimulator of skeletal muscle protein anabolism and important for maintenance of muscle mass and overall muscle health. Circulating amino acid kinetics and acute skeletal muscle protein anabolic responses have been extensively evaluated following ingestion of dairy proteins such as casein and whey protein isolate [1,2,3,4,5]. Though these data have provided fundamental information in understanding how muscle responds to protein, it is less generalizable to the community since most dietary protein sources contain a mixed-macronutrient profile, contain many micronutrients within their matrix, and are more complex during digestion.

Recent protein metabolism studies have evaluated blood amino acid kinetics and muscle anabolic responses to protein-enriched, nutrient-complex foods such as beef, egg, and pork [6,7,8,9,10,11,12,13,14] and as a result, have demonstrated unique amino acid and protein anabolic responses. For example, consumption of 18 g of protein from whole egg after a bout of exercise increased protein synthesis more so than egg whites in spite of similar post absorptive plasma leucine levels [14]. This suggests protein-dense whole foods have utility to promote protein anabolism not simply predicted by the amount of protein or level of aminoacidemia, which is in contrast to what has been observed with isolated protein products [5]. Therefore, there is a continued need to characterize whole food products to identify high quality protein sources that encourage human health.

To our knowledge, no studies have evaluated the amino acid pattern in plasma or muscle anabolic response to cheese ingestion. Cheddar cheese, is a low carbohydrate, high-fat, protein-rich food that is a regular dietary component of the U.S. diet [15]. Cheddar cheese has a well characterized amino acid profile with a high content of leucine (~10%) and is considered low glycemic. Moreover, the protein in Cheddar cheese is partially hydrolyzed due to aging/ripening [16], and therefore is likely to speed up digestion and promote the appearance of amino acids in the circulation [4,17]. Cheddar cheese is also composed of many other underappreciated nutrients within its food matrix [18] that are beneficial for human health and could further enhance protein anabolism.

Therefore, the primary purpose of this study was to characterize the amino acid response following 65 g (20 g protein) of Cheddar cheese, an amount of protein capable of increasing blood amino acid levels from a whole dairy product [7,9,19]. In addition, to gain insight on the protein anabolic processes in skeletal muscle, we evaluated mTORC1 localization and cellular signaling following cheese ingestion, given that mTORC1 intracellular signaling is highly responsive to acute protein intake particularly to sources that are rich in leucine [20,21]. We also complimented mTORC1 signaling with a unique ‘omics approach of ribosome profiling [22] to capture key information regarding which mRNAs are translated after cheese ingestion. Finally, to provide context in comparison to a well-described whole food, we conducted a within subject crossover study comparing to an isonitrogenous amount of milk [19]. We hypothesized that a single dose of Cheddar cheese in young male and female adults, equivalent to 20 g of protein, would acutely increase the blood branched-chain amino acids (particularly leucine) and induce a translational program characterized by mTORC1 signaling.

## 2. Methods

### 2.1. Subjects

Twenty-four young male (*n* = 12) and female (*n* = 12) subjects participated in this study (Table 1; 27 ± 4 years; BMI 23.1 ± 3.5 kg/m^2^). Interested subjects were notified of the study through posted flyers on campus and in areas around the university and were also contacted through the University of Utah PEAK Health and Fitness registry. Subjects were screened (self-report) based on the following exclusion criteria: history of cardiovascular disease, endocrine or metabolic disease (e.g., hypo/hyperthyroidism, diabetes), kidney disease or failure, liver disease, respiratory disease (acute upper respiratory infection, chronic lung disease), stroke with motor disability, use of anticoagulant therapy (e.g., Coumadin, heparin) including aspirin and fish oils within 7 days (d) of the first metabolic experiment, elevated systolic blood pressure > 150 or a diastolic blood pressure > 100, smoking, recent anabolic or corticosteroids use (within 12 weeks of first biopsy), pregnancy, lactose intolerance, and irregular menstruation. Enrolled participants read and signed the informed consent document, which was approved by the University of Utah Institutional Review Board (IRB #110963) and in agreement with the Declaration of Helsinki. This study is registered at clinicaltrials.gov (NCT04660877).

### 2.2. Experimental Design

After enrollment, participants completed baseline testing which included a dietary assessment, body composition and habitual activity levels. Body composition (lean and fat mass) was assessed using a Bod Pod instrument (conducted prior to Metabolic Study #1). Physical activity was tracked for a 7 days period between the Metabolic Study visits. Additionally, a 3 d daily dietary record (ASA24) was recorded before each Metabolic Study visit. The daily dietary record was averaged between all recorded days and reported in Table 1.

**Figure 1:**
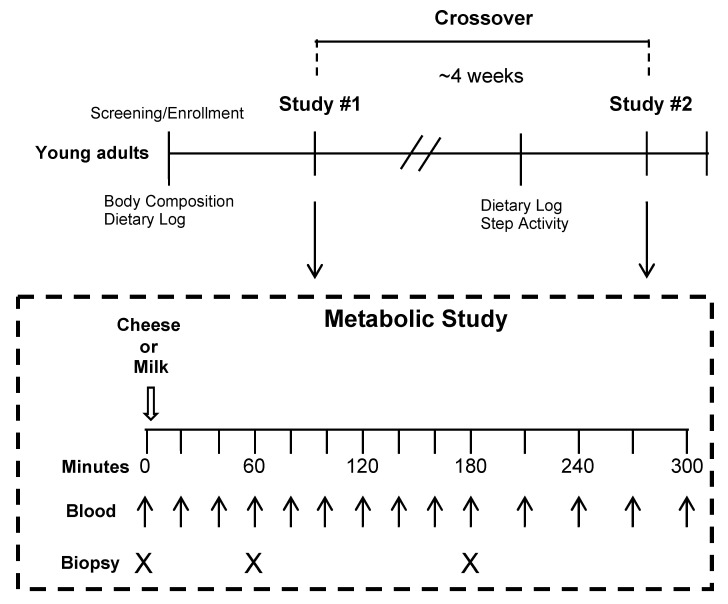
Overview of the crossover study experimental design.

Each subject took part in two metabolic studies (Figure 1) with each designed to test the acute blood and muscle response to an ingested amount of either Cheddar cheese or milk matched for protein (Table 2). Approximately, one month after the first experiment (Metabolic Study #1), the participant completed the second experiment (Metabolic Study #2) which was exact in design and at the same time of day as the first study but the participant ingested the alternate food product. Prior to each of the metabolic studies, the participant ate a standardized research meal the night before the study and refrained from intense physical activity for 48 h. The morning of the metabolic studies, the participant arrived at the clinical research center after a ~10 h fast. A catheter was then placed in the participants’ arm for blood sampling. Next, the participant underwent a baseline vastus lateralis skeletal muscle biopsy (0 min) using a modified version Bergström muscle biopsy technique [23]. Following the baseline muscle biopsy, the participant consumed either Cheddar cheese (65 g) or milk (370 mL; 2%; Fairlife) each amounting to 20 g of protein. The Cheddar cheese was processed at Glanbia Nutritionals, aged to one month, and frozen into batches distributed monthly by the sponsor as needed. The amino acid profile of low-fat Cheddar cheese and 2% Fairlife milk can be found in Supplemental Table S1. Subsequent muscle biopsies occurred 60 and 180 min on the same thigh after product ingestion which is an ideal timeframe to capture mTORC1 signaling and mRNA translational events following protein-enriched nutrient ingestion [24,25]. Blood sampling occurred before and periodically after ingestion of the products (up to 300 min). Blood samples were taken every 20 min during the first 3 h and then every 30 min for the last 2 h. Therefore, there were a total of 14 blood draws and 3 muscle biopsies for each Metabolic Study visit. The starting thigh for muscle biopsies for the first Metabolic Study was randomized for each subject and balanced with the second Metabolic Study (left leg then right or right leg then left). Muscle samples were frozen in liquid nitrogen (for immunoblotting and ribosomal profiling) or prepared in O.C.T. (Optimal Cutting Temperature) and frozen in liquid nitrogen-cooled isopentane for the immunohistochemical assessment.

### 2.3. Blood Analyses

Blood samples were collected in EDTA (Ethylenediaminetetraacetic) vacutainer collection tubes and immediately placed on ice. Samples were centrifuged (2500 rpm, 10 min) and plasma was collected and frozen at −80 °C until later analysis. Plasma was processed for essential and non-essential amino acids using the EZ:Faast Amino Acid Kit (Phenomenex; Cat #KG0-7165) and analyzed using GCMS analysis in collaboration with the institution’s Metabolomics Core. Essential amino acids included detection of leucine, isoleucine, valine, threonine, methionine, phenylalanine, lysine, histidine, and tryptophan. Non-essential amino acids included detection of alanine, glycine, serine, proline, asparagine, glutamate, glutamine, and tyrosine. Samples were also immediately assessed for glucose (YSI) at the time of the study and later assessed for insulin (Human Insulin ELISA, Millipore Sigma, Burlington, MA, USA; EZHI-14K) and non-esterified fatty acids (NEFA-HR; Wako Chemicals, Richmond, VA, USA) in replicate using commercially available kits. Insulin and free fatty acids were determined at select time points (baseline, 20, 40, 80, 140, 210, and 300 min).

### 2.4. Skeletal Muscle Immunoblotting

Approximately 30 mg of tissue at each biopsy time point for Cheddar cheese and milk interventions was homogenized 1:10 (wt/vol) using a glass tube and mechanically-driven pestle grinder in an ice-cold buffer containing 50 mM Tris (pH 7.5), 250 mM mannitol, 40 mM NaF, 5 mM pyrophosphate, 1 mM EDTA, 1 mM EGTA, and 1% Triton X-100 with a protease inhibitor cocktail. Homogenates were centrifuged for 10 min at 4 °C. After centrifugation, the supernatant was collected and the protein concentration was determined using a modified Bradford protein assay and measured by a spectrophotometer (EPOCH; BioTek, Winooski, VT, USA).

Thirty micrograms of protein from muscle homogenate was separated via polyacrylamide gel electrophoresis, transferred onto a polyvinylidene difluoride membrane (PVDF), and incubated with primary and secondary antibodies. PVDF Membranes were imaged on a ChemiDoc XRS (Bio-Rad, Hercules, CA, USA) and quantified with Image lab software (Bio-Rad). The primary antibodies were purchased from Cell Signaling Technology and were the following: phospho-S6K1, Thr389, 1:1000, #9205; phospho-ribosomal protein S6, RPS6, Ser240/244, 1:1000, #2215; phospho-AS160, Ser588, 1:1000, #8730; phospho-GSK-3β, Ser9, 1:1000, #9336; phospho-Akt, Ser473, 1:1000, #9271. Secondary antibody (HRP Anti-Rabbit, #SC2004, 1:2000) was purchased from Santa Cruz Biotechnology. Phosphorylation of these proteins were normalized to Ponceau-S staining and reported as fold change from baseline.

### 2.5. Skeletal Muscle Immunohistochemistry

Muscle was sectioned into 8 µm cross-sections, mounted on slides in −25 °C, then left to air-dry overnight, and stored at −20 °C. Immunofluorescent staining was used to detect mTORC1 (Cell Signaling Technology, #2983, 1:100), the lysosomes (LAMP2: Abcam, #ab25631, 1:100), and the membrane (WGA: Fisher Scientific, #W32466, 1:50) as demonstrated by others [26,27,28,29]. Briefly, tissue was fixed in acetone (10 min), and the following blocking steps were performed: (1) endogenous peroxidases: 3% H_2_O_2_ for 7 min, (2) Non-Specific Binding Sites: 5% goat serum, Vector Labs #S-1000 with 0.3% Triton-X for 1 h, and (3) Avidin/Biotin: Vector Labs #SP-2001 according to manufacturer’s instructions. WGA was added (5 min), and mTOR and LAMP2 were incubated on the slide overnight. Secondary antibody for LAMP2 was performed using Alexa Fluor 488 Tyramide SuperBoost (Invitrogen, #B40932, according to manufacturer’s instructions), while secondary for mTOR was on Cy3 (Jackson ImmunoResearch, #711-165-152; 1:500) for 1 h. Finally, slides were mounted, cover slipped (Vectashield with DAPI, Vector Labs, #H-1200), and stored in the fridge until imaged (within 1 month of staining).

Images were taken using a Leica SP8 White Light laser confocal microscope equipped with automated stage, and Nikon NIS-Elements multi-platform acquisition software. At least 9 images (16 bit) were taken at 40X/1.3 magnification with oil immersion, with each image capturing ~5 muscle fibers per image in high detail at each time point, analyzing a total of ~45 muscle fibers per subject per time point, for each product consumed. When looking at events detected above threshold (set with help of combinations of positive and negative controls) of mTOR and LAMP2, anything not within 80% of the average was not used. The number of objects/events per channel times the average area covered by each object gave us the total area per channel. As previously described [26], Mander’s overlap coefficient of colocalization was employed (k1 for mTOR/LAMP2; k2 for mTOR/WGA) to quantify the cellular overlap of these proteins, and this was performed in NIS-Elements for mTOR/LAMP2 and mTOR/WGA.

### 2.6. Ribosomal Profiling

Muscle samples at each time point (0, 60, 180 min) from Cheddar cheese and milk studies were assessed from a subset of subjects (4 subjects, 2 M, 2 F). Traditional RNA-Seq captures total mRNA abundance within a tissue sample, while the emerging technique of Ribo-Seq allows the capture of ribosome protected fragments (RPF) measuring translational activity in a transcript-specific manner [30,31]. Polysome complexes were isolated, and unprotected mRNA digested with RNase I, and the ribosome protected mRNA footprints were analyzed by RNA-Sequencing methods as previously described by our group [22] with the exception that rRNA was removed from the RPF samples using the NEBNext rRNA Depletion kit and libraries were size selected by polyacrylamide gel electrophoresis on 6% native gels. Libraries were then sequenced on an Illumina Novaseq 6000 instrument. Raw sequence data can be obtained from the National Center for Biotechnology Information Gene Expression Omnibus repository entry GSE163279.

Uniquely mapping sequences were identified by alignments using bowtie to Reference Sequence database (RefSeq) mRNA entries obtained from the University of California, Santa Cruz browser (Hg38 human genome reference assembly) in which all mRNAs derived from the same gene were reduced to a single entry corresponding to the longest isoform. Normalization factors based on the trimmed mean of M-values were determined by using the calcNormFactors function of the Bioconductor package edgeR [32]. Dispersion estimates were obtained prior to likelihood ratio tests (glmFit and glmLRT functions of edgeR) to determine significance of the log_2_ fold change in RPFs or RNA for all transcripts with ≥1 count/million in all samples. Differences were considered significant if the false discovery rate was ≤0.05. Pearson’s product-moment correlation coefficients were calculated.

Ingenuity Pathway Analysis was performed to determine significantly altered pathways informed by the translation changes at each time point for the two respective protein sources. mTOR pathway volcano plots used all of the molecules within the top 3 pathways (‘EIF2 Signaling’, ‘Regulation of eIF4 and p70S6K Signaling’, ‘mTOR Signaling’) in either cheese or milk for comparison, yielding presentation of the translation for 202 total transcripts, at 3 contrasts (60 vs. 0 min translation f.c.; 180 vs. 0 min translation f.c. and 180 vs. 60 min translation f.c.).

### 2.7. Statistical Analyses

Subject characteristics were compared between males and females using a t-test. Because there were no notable differences between males and females in major outcomes (i.e., blood amino acids), subjects were pooled and all comparisons (Plasma NEFA, Insulin, Amino Acids, Immunoblotting, and IHC colocalization) were analyzed using a 2-Way ANOVA with repeated measures for product and time. When appropriate after a significant interaction was detected, Sidak’s multiple comparisons post-hoc test was used to identify differences from baseline or between protein products at a given time point. For all analyses, differences were considered statistically significant at *p* < 0.05. All statistical calculations and graphs were completed using GraphPad Prism (v8).

## 3. Results

### 3.1. Subject Characteristics

A total of 24 young adult participants completed both trials of this study. This was made up of 12 males and 12 females (Table 1). As expected, men had greater height, body weight, and had more lean mass than females (*p* < 0.05). The men also had less daily step activity than the females (*p* < 0.05). There were no differences between the sexes in age, BMI, fat mass, body fat % or daily protein intake.

### 3.2. Blood Insulin, Glucose and Non-Esterified Fatty Acids

**Figure 2:**
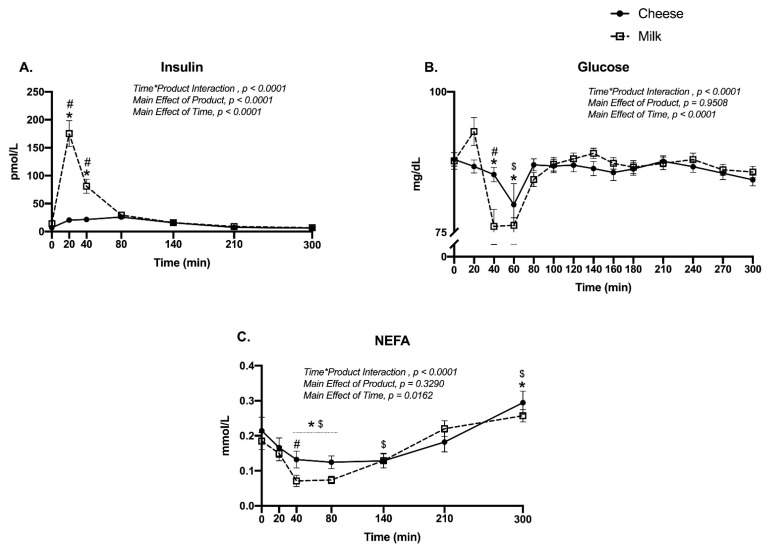
Plasma (**A**) insulin (pmol/L), (**B**) glucose (mg/dL), and (**C**) non-esterified fatty acids (mmol/L) in the fasted state (0 min) and over a 300 min time period following the ingestion of either cheese (solid line) or milk (dotted line) in men and women (*n* = 24). Different from baseline (0 min) for milk (*) and cheese (^$^), *p* < 0.05. ^#^, Different between groups at the specific time point, *p* < 0.05.

Milk induced a rapid spike in insulin 20 min after ingestion (2-Way ANOVA: Time*Product Interaction, *p* < 0.0001; Sidak’s multiple comparisons test, Milk different from baseline and from cheese at 20 and 40 min, *p* < 0.0001) while cheese consumption did not significantly change insulin at any time point (Figure 2A). Blood glucose decreased at 60 min following ingestion of either product, but this decrease occurred earlier for milk (40 min; Time*Product Interaction, *p* < 0.0001) and was significantly lower than cheese (Figure 2B). Similarly, NEFA levels decreased after ingestion of either Cheddar cheese or milk, (Time*Product Interaction, *p* < 0.0001), but this response was further decreased for milk compared to Cheddar cheese (Sidak’s multiple comparisons test, Cheese vs. milk 40 min post, *p* = 0.023). Additionally, NEFA levels were significantly elevated in response to both protein sources by 300 min, in comparison to baseline NEFA values (Sidak’s multiple comparisons test, Cheese: *p* = 0.003; Milk: *p* = 0.011) (Figure 2C).

### 3.3. Plasma Branched-Chain, Essential and Non-Essential Amino Acids

**Figure 3:**
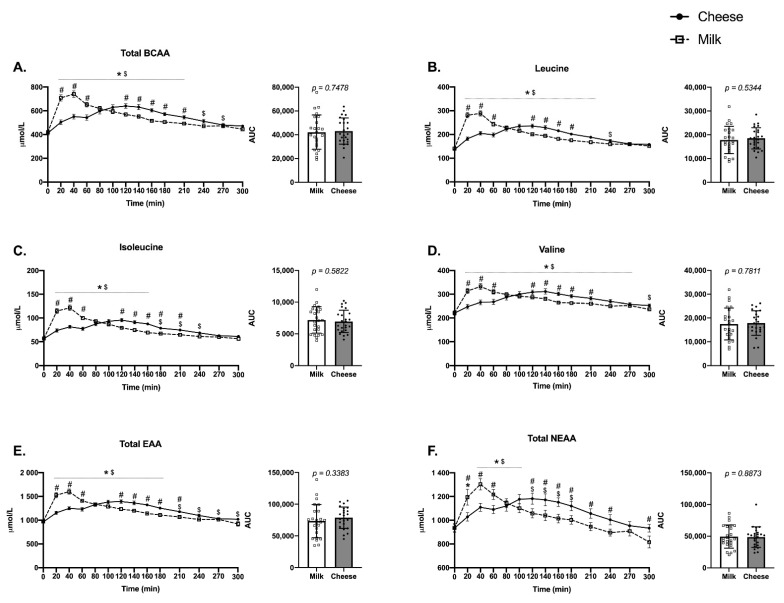
Plasma levels (μmol/L) of (**A**) branched-chain amino acids (Total BCAA), (**B**) leucine, (**C**) isoleucine, (**D**) valine, (**E**) essential amino acids (Total EAA), and (**F**) non-essential amino acids (Total NEAA) in the fasted state (0 min) and over a 300 min time period following the ingestion of either cheese (solid line) or milk (dotted line) in men and women (*n* = 24). Different from baseline (0 min) for milk (*) and cheese (^$^), *p* < 0.05. ^#^, Different between groups at the specific time point, *p* < 0.05. Units are in micromolar (μM). Note: Total EAA (**E**) does not include the BCAAs.

Total branched-chain amino acids (BCAAs) increased with different kinetics in response to ingestion of the respective products (Time*Product Interaction, *p* < 0.0001) (Figure 3A). After milk, BCAAs returned to baseline by 240 min post, and cheese maintained higher BCAA levels out to 270 min. Milk induced significantly higher BCAA levels than cheese from 20 to 60 min post ingestion, and decreased gradually towards baseline as cheese induced significantly higher BCAA in plasma than milk between 120 and 210 min (Sidak’s, *p* < 0.05). Plasma leucine exhibited a similar response as total BCAAs (Time*Product Interaction, *p* < 0.0001) (Figure 3B), with both products increasing leucine levels out to 210 min and with cheese elevating leucine levels slightly longer to 240 min (Sidak’s, *p* < 0.05). The leucine response occurred to a greater magnitude for milk from 20 to 60 min while cheese induced higher leucine levels (vs. milk) from 120 to 180 min. Plasma isoleucine (Time*Product Interaction, *p* < 0.0001) (Figure 3C) increased out to 160 min for milk while cheese increased isoleucine levels out to 240 min. Milk had a greater isoleucine response compared to cheese from 20 to 60 min while cheese had a greater plasma isoleucine response than milk from 120 to 210 min (Sidak’s, *p* < 0.05). Plasma valine (Time*Product Interaction, *p* < 0.0001) (Figure 3D) increased over the 300 min time course for cheese and out to 270 min for milk. This response was greater for milk at 20–60 min while the cheese induced a greater valine level than milk from 120 to 210 min (Sidak’s, *p* < 0.05). Total essential amino acids (EAA) increased above baseline for milk out to 180 min while cheese increased total EAA out to 300 min (Time*Product Interaction, *p* < 0.0001) (Figure 3E). Plasma EAA were higher for milk from 20 to 60 min (compared to cheese) while EAA were higher for cheese from 120 to 210 min (vs. milk). Non-Essential amino acids (NEAA) (Figure 3F) increased above baseline for milk from 20 to 100 min while NEAA were elevated above baseline from 40 to 180 min for cheese (Time*Product Interaction, *p* < 0.0001). Milk induced a greater NEAA response at 20–60 min while cheese induced a greater response than milk from 120 to 300 min (except at 270 min). Despite differences in amino acid kinetics between the products, the area under the curve over 5 h for total BCAA, leucine, isoleucine, valine, total EAA, and total NEAA were not different between cheese and milk products (Figure 3A–F).

### 3.4. Muscle mTORC1 Signaling and Localization

**Figure 4:**
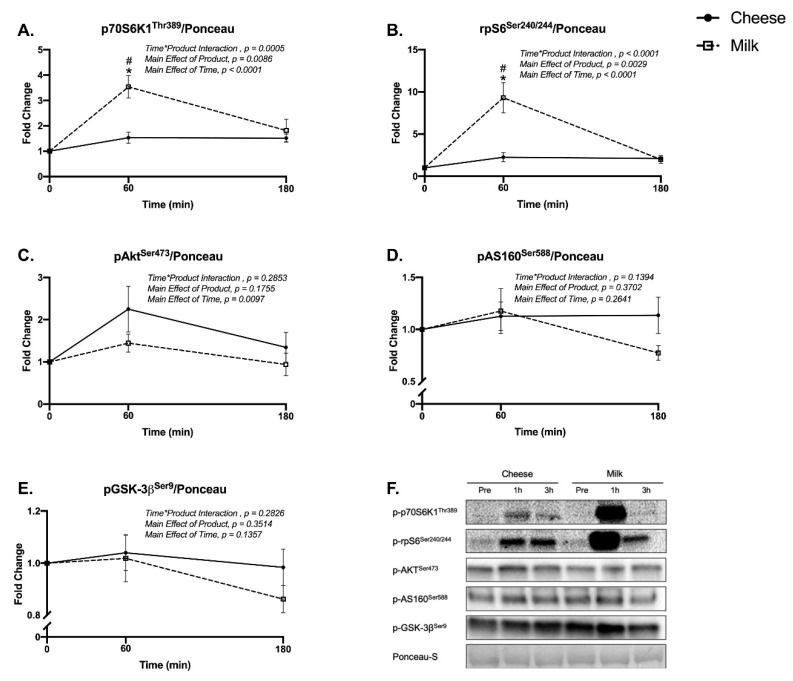
Skeletal muscle protein expression using immunoblotting for (**A**) p70S6K1(Thr389), (**B**) rpS6(Ser240/244), (**C**) Akt(Ser473), (**D**) AS160(Ser588), and (**E**) GSK-3β(Ser9) in the fasted state (0 min) and at 60 and 180 min following the ingestion of either cheese (solid line) or milk (dotted line) in men and women. Panels (**A**,**B**) are data for *n* = 24 while for (**C**–**E**) only *n* = 8 (4 M, 4 F) were analyzed. Panel (**F**) are representative images of immunoblotting. Phosphorylated protein levels were normalized to Ponceau-S. Different from baseline (0 min) for milk (*), *p* < 0.05. ^#^, Different between groups at the specific time point, *p* < 0.05.

Phosphorylated p70S6K(Thr389) (Time*Product Interaction, *p* = 0.0005) and phosphorylated rpS6(Ser240/244) (Time*Product Interaction, *p* < 0.0001) increased above baseline and were increased to a greater extent for milk at 60 min post ingestion compared to cheese (Sidak’s multiple comparisons test, *p* < 0.0001 for p70S6K and rpS6K) (Figure 4A,B). Phosphorylated Akt(Ser473) was significantly elevated 60 min post ingestion as a result of cheese or milk with no difference between cheese and milk (2-Way ANOVA: Main Effect of Time, *p* = 0.0097) (Figure 4C). There were no significant differences detected for phosphorylated AS160(Ser588) or phosphorylated GSK-3β(Ser9) (Figure 4D,E). Figure 4F is representative immunoblotting images for the phosphorylated proteins.

**Figure 5:**
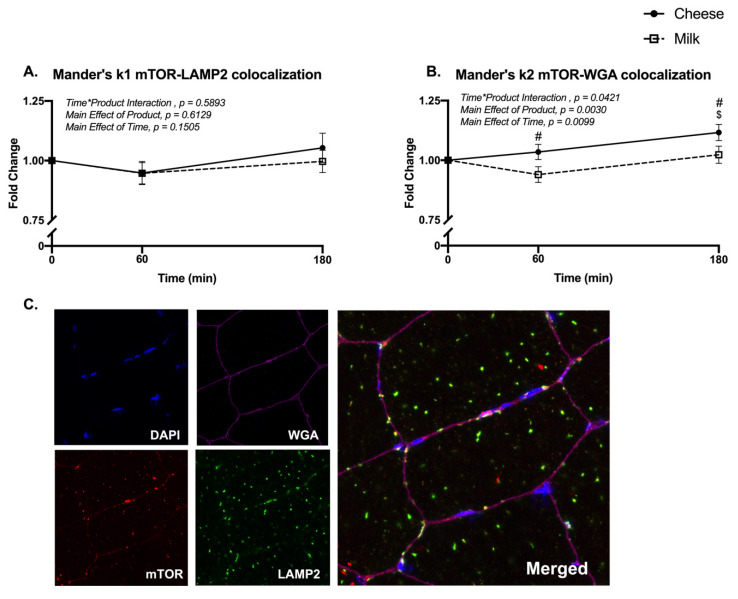
Skeletal muscle mTOR colocalization using immunohistochemistry. Panel (**A**) represents Mander’s k1 mTOR-LAMP2 colocalization and (**B**) Mander’s k2 mTOR-WGA colocalization at baseline (0 min) and at 60 and 180 min following the ingestion of either cheese (solid line) or milk (dotted line) in men and women (*n* = 24). Panel (**C**) are representative images using immunohistochemistry. Different from baseline (0 min) for cheese (^$^), *p* < 0.05. ^#^, Different between groups at the specific time point, *p* < 0.05.

Using immunohistochemistry to fluorescently label and measure the spatial distribution of mTOR, we did not detect changes to the colocalization of mTOR with the lysosomal protein, LAMP2 (Figure 5A). However, mTOR colocalization with the sarcolemma (WGA) was different between groups at 60 and 180 min and increased at 180 min only after cheese ingestion (Time*Product Interaction, *p* = 0.042; Sidak’s multiple comparisons test, *p* = 0.003) (Figure 5B). Representative images for DAPI, WGA, mTOR, LAMP2 and the overlay are found in Figure 5C.

### 3.5. Ribosomal Profiling

**Figure 6:**
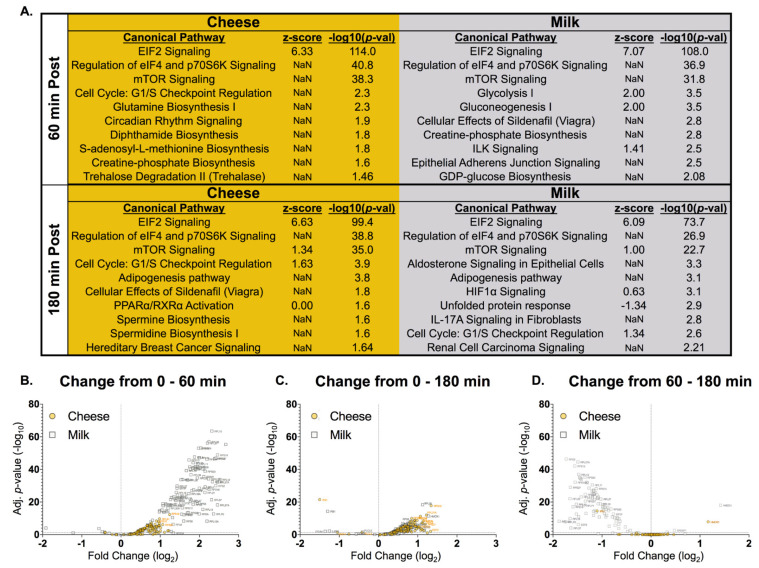
Skeletal muscle analysis of translated mRNAs assessed using ribosomal sequencing before and after cheese or milk ingestion in men and women (*n* = 4; 2 M, 2 F). (**A**) Canonical pathways identified by ingenuity pathway analysis for cheese and milk at 60 and 180 min and (**B**) Volcano plot of the change in translated mRNAs for cheese (orange squares) or milk (gray squares) from 0 to 60 min, (**C**) 0 to 180 min, and (**D**) 60 to 180 min post ingestion.

A subset of subjects’ muscle samples (*n* = 4) was used for ribosomal profiling. Ribosomal profiling captures ribosome protected mRNA fragments to measure active translation of specific transcripts using RNA sequencing libraries. Both cheese and milk altered the same top 3 Canonical Pathways related to mTORC1 signaling (IPA: EIF2 Signaling, Regulation of eIF4 and p70S6K Signaling, mTOR Signaling) (Figure 6A) at both 60 and 180 min, while only milk activated glucose metabolism-related pathways (Glycolysis I, Gluconeogenesis I) 60 min post ingestion. Next, we created a volcano plot for the significantly altered transcripts from within the top 3 Canonical Pathways for cheese and milk respectively, representative of all translation changes under control of mTORC1 signaling. As a result, we demonstrated a significant and dramatic milk-induced (in comparison to cheese) translational response from 0 to 60 min for these mTORC1 mediated molecules (Figure 6B). This response for milk was reduced at 0–180 min after ingestion while cheese-induced translation of mTORC1 molecules was maintained at similar levels as was observed at 60 min (Figure 6C). Moreover, translation changes across the 60–180 min time period (Figure 6D) highlight the observation that stimulation of mTORC1 pathway is reduced at 180 min after milk ingestion but persists with cheese.

## 4. Discussion

Our current understanding of amino acid kinetics and subsequent skeletal muscle anabolism following protein intake has been informed by isolated protein sources (and often in liquid form) such as whey [33], casein [34], soy [5], and leucine-enriched EAAs [35]. Recently, the study of solid protein-enriched whole foods, of which the food matrix can greatly alter protein digestion and absorption kinetics and the subsequent muscle anabolic signature, is a valuable next step in studying the impact of dietary interventions on muscle health and disease [36]. The purpose of our study was to examine the response to 20 g of protein from Cheddar cheese on plasma amino acids, free fatty acids, insulin, and glucose and the subsequent skeletal muscle mTORC1 signaling and mRNA translational response. To better contextualize the results of Cheddar cheese ingestion with what is known in the field, we utilized a crossover design with comparison to milk, a highly studied protein source with a well-characterized absorption profile and muscle anabolic response [19,37]. The results from this study indicate that Cheddar cheese had a slow, yet persistent amino acid circulation appearance and subsequent skeletal muscle mTORC1 signaling and mRNA translation response when compared with the quick absorption and potent but short-lived mTORC1 stimulation induced by milk. At the studied dosage, Cheddar cheese did not induce a plasma insulinogenic or muscle glycemic response, a known effect of milk [19,38], suggesting Cheddar cheese may be an interesting food choice for dietary strategies geared to promote muscle protein anabolism yet requiring strict glycemic control.

The primary finding of this study was that consumption of Cheddar cheese (65 g) amounting to 20 g of protein promoted a delayed, yet sustained plasma amino acid concentration over 5 h, compared with the acute and potent appearance of circulating amino acids induced by milk proteins. Even though the amounts of protein and leucine were similar between the products, milk resulted in a more rapid and robust amino acid response likely driven by the whey protein component (20% whey, 80% casein in bovine milk vs. 100% casein in Cheddar cheese). Nonetheless, we found it interesting that there was no difference in the total circulating amino acids across the entire 5 h time course between cheese and milk. Thus, although the solid food matrix of cheese and protein composition may slow the digestion and absorption of protein and, subsequently, amino acid release into the circulation, when matched for protein, cheese and milk have similar total plasma amino acid availability. Casein hydrolysate, the form present in Cheddar cheese, has shown to result in a greater appearance in circulating levels of leucine compared with intact casein [4,17,39]. Though difficult to compare to a liquid casein beverage, the plasma leucine appearance data following 20 g Cheddar cheese protein ingestion demonstrated a slower plasma leucine appearance rate and magnitude compared to a similar amount of isolated casein hydrolysate [4] suggesting that the complex matrix of cheese may delay the release of amino acids into circulation [40]. It is currently unknown if a longer aged Cheddar cheese may speed the circulating appearance of amino acids. However, when compared with other solid, protein-dense foods, such as pork [6], cooked egg [14], and steak [13], Cheddar cheese aged to one month produced a similar plasma appearance, magnitude, and sustained amino acid availability response.

We also measured muscle mTORC1 activation using three different approaches with the cumulative result of these assessments demonstrating that anabolic signaling tracked closely with circulating amino acids for each product and with milk demonstrating a more robust mTORC1 signaling response early after intake (1 h). This is logical since essential amino acids, especially leucine, along with insulin, which also peaked prior to 1 h, are stimulators of mTORC1-mediated protein synthesis [41,42]. It is likely that the insulin response from milk, combined with the quickly absorbed leucine, synergized to enhance mTORC1 signaling as noted by the magnitude of p70S6K and rpS6 activation [42]. It is well known that anabolic cues such as insulin, mechanical stimulation, and amino acid ingestion stimulate mTORC1 and its downstream effectors (e.g., S6K1) to enhance translation initiation [43]. While our measurement of mTORC1 signaling was limited to a 3 h time course (based on other protein-dense whole food studies [6,13,14]), we found it noteworthy that mTORC1 activation following Cheddar cheese ingestion persisted at 3 h (and possibly beyond) in accordance with the plasma amino acid appearance and as supported by the mTORC1 localization data and the ribosomal profiling of translated mRNAs under control of mTORC1 signaling. It is unclear how a sustained circulation of amino acids following Cheddar cheese intake may impact muscle protein accretion. Whole foods that are slow digesting (in comparison to commonly studied dairy protein drinks) may have utility in sustaining the free amino acid pool so that they have a longer window to synergize with other anabolic cues such as exercise, or by offsetting protein breakdown to enhance net protein balance when combined with the acute stimulus of a faster digesting protein source [3,44]. For example, drinking a small glass of milk with cheese, may result in a greater net protein balance over several hours in comparison to a bolus of milk alone, because of the ceiling for acute anabolic activation and subsequent oxidation of excess amino acids (coined the ‘muscle-full effect’) thereby limiting the anabolic benefit of the beverage [38,44,45]. There is a similar underlying premise behind the recommendation of ingesting casein (a major component of cheese) prior to bedtime to enhance exercise adaptations [46,47,48].

Another interesting observation about the acute response to Cheddar cheese intake (in contrast to milk) in this study, was that cheese did not observably increase circulating insulin or translation of muscle mRNAs related to glycolytic pathways at any time point we measured after ingestion. Therefore, if a dietary intervention requires strict glycemic control, such as for individuals with diabetes [49] or requires adhering to a ketogenic diet [50], cheese may be a valuable protein food source to keep on the menu. The two most likely reasons for why cheese and milk ingestion had different circulating insulin responses, are that (a) milk contains higher levels of carbohydrates and (b) milk induced an early spike in circulating serum leucine (compared with cheese), which stimulates endogenous insulin release [51]. In addition to being less glycemic, the general public’s health opinion of cheese should be re-examined since regular consumption of cheese does not appear to influence LDL or HDL levels despite the characteristically high fat content [52]. Though Cheddar cheese does incorporate a significant portion of its calories from fat, fat does not appear to influence muscle protein anabolism [53], and may even synergize with protein to promote a greater anabolic response [14,36]. However, because of the extra calories associated with fat as compared to other macronutrients, individuals who must restrict their calories may benefit from reduced fat cheese.

## 5. Conclusions

In summary, Cheddar cheese provided a slow and sustained appearance of circulating amino acids and subsequent activation of mTORC1 signaling when compared to milk matched for protein (and leucine) content. Also, Cheddar cheese at the amount consumed in this study did not noticeably increase circulating insulin or induce a muscle glycemic response in contrast with milk. Overall, low fat Cheddar cheese should be considered as a protein-dense food choice given its high leucine content, ability to sustain amino acid levels and promote protein anabolism and, especially, considering its low glycemic properties. Future studies are needed to examine muscle protein accretion in response to daily Cheddar cheese ingestion when combined with habitual exercise.

## Figures and Tables

**Table 1:** Subject Characteristics.

	Pooled	Male	Female
Sample Size (*N*)	24	12	12
Age (year)	27 ± 4	27 ± 4	26 ± 4
Height (cm)	175 ± 8	181 ± 5 *	169 ± 7
Body Mass (kg)	71 ± 14	80 ± 12 *	63 ± 10
BMI (kg/m^2^)	23.1 ± 3.5	24.6 ± 3.8	21.9 ± 2.8
Lean Mass (kg)	57.5 ± 11.9	67.9 ± 8.7 *	48.1 ± 4.9
Fat Mass (kg)	13.5 ± 7	12.5 ± 8	14.9 ± 6
Body Fat (%)	18.7 ± 8	14.8 ± 8	22.8 ± 6
Daily Protein Intake (g/kg/day)	1.32 ± 0.49	1.40 ± 0.61	1.25 ± 0.35
Steps/Day	8798 ± 3444	7364 ± 2845 *	10,122 ± 3514

Mean ± SD, * Different from Female (*p* < 0.05).

**Table 2:** Nutrient content of experimental products.

	Cheddar Cheese	2% Fairlife Milk
Amount	65 g	370 mL
Protein (g)	20	20
Leucine (g)	1.97	1.98
Fat (g)	10	7.5
Carbohydrates (g)	0	9
Calories (kcal)	170	183

## Data Availability

Raw sequence data can be obtained from the National Center for Biotechnology Information Gene Expression Omnibus repository entry GSE163279.

## Supplementary Materials

The following are available online at https://www.mdpi.com/2072-6643/13/2/614/s1, Table S1: Amino acid composition of Cheddar cheese and milk.
