# Supplementary material for: Acute Effects of Cheddar Cheese Consumption on Circulating Amino Acids and Human Skeletal Muscle
Source: Nutrients. 2021 Feb 13;13(2):614. doi: 10.3390/nu13020614 (PMC7917914; doi:10.3390/nu13020614)
Supplement: Supplementary file 1 [file nutrients-13-00614-s001.pdf]

Supplemental Table S1. Amino acid composition of Cheddar cheese and milk

	**Cheddar Cheese**	**2% Milk**
	g/20g protein	g/20g protein
Alanine	0.582	0.640
Arginine	0.768	0.710
Aspartic Acid	1.438	1.544
Cystine	0.065	0.150
Glutamic Acid	4.540	4.240
Glycine	0.384	0.384
Histidine	0.616	0.582
Isoleucine	1.008	1.036
Leucine	1.972	1.988
Lysine	1.654	1.702
Methionine	0.540	0.520
Phenylalanine	1.114	1.016
Proline	2.200	1.942
Serine	1.186	1.122
Threonine	0.772	0.876
Tyrosine	1.228	1.102
Valine	1.426	1.340

Note: Tryptophan detection not performed
